# Retinoid Homeostasis and Beyond: How Retinol Binding Protein 4 Contributes to Health and Disease

**DOI:** 10.3390/nu14061236

**Published:** 2022-03-15

**Authors:** Julia S. Steinhoff, Achim Lass, Michael Schupp

**Affiliations:** 1 Charité—Universitätsmedizin Berlin, Corporate Member of Freie Universität Berlin and Humboldt-Universität zu Berlin, Institute of Pharmacology, Cardiovascular Metabolic Renal (CMR)-Research Center, 10115 Berlin, Germany; julia.steinhoff@alumni.charite.de; 2 Institute of Molecular Biosciences, NAWI Graz, University of Graz, Heinrichstraße 31/II, A-8010 Graz, Austria; achim.lass@uni-graz.at; 3 Field of Excellence BioHealth, University of Graz, Heinrichstraße 31/II, A-8010 Graz, Austria

**Keywords:** RBP4, vitamin A, retinoids, retinol transport, retinoid homeostasis, metabolism, liver

## Abstract

Retinol binding protein 4 (RBP4) is the specific transport protein of the lipophilic vitamin A, retinol, in blood. Circulating RBP4 originates from the liver. It is secreted by hepatocytes after it has been loaded with retinol and binding to transthyretin (TTR). TTR association prevents renal filtration due to the formation of a higher molecular weight complex. In the circulation, RBP4 binds to specific membrane receptors, thereby delivering retinol to target cells, rendering liver-secreted RBP4 the major mechanism to distribute hepatic vitamin A stores to extrahepatic tissues. In particular, binding of RBP4 to ‘stimulated by retinoic acid 6’ (STRA6) is required to balance tissue retinoid responses in a highly homeostatic manner. Consequently, defects/mutations in RBP4 can cause a variety of conditions and diseases due to dysregulated retinoid homeostasis and cover embryonic development, vision, metabolism, and cardiovascular diseases. Aside from the effects related to retinol transport, non-canonical functions of RBP4 have also been reported. In this review, we summarize the current knowledge on the regulation and function of RBP4 in health and disease derived from murine models and human mutations.

## 1. Introduction to RBP4

The term vitamin A refers to retinol and its bioactive derivatives, which are known to mediate a plethora of physiological functions. Essential for humans [1], vitamin A is involved in processes including vision, immune cell function, reproduction, embryonic development, and the regulation of cell proliferation and differentiation [2,3,4,5]. Retinol, being the most abundant form of vitamin A in the bloodstream, requires a carrier protein due to its lipophilic nature. In 1968, RBP4 was identified as the specific retinol transporter in the circulation, which is reflected in the name ‘retinol binding protein 4’ (RBP4, also known simply as RBP and encoded by the *Rbp4* gene ). Injecting radio-labeled retinol and subsequently extracting retinol that had been bound to a carrier protein from plasma led to the discovery of RBP4 [6]. Human RBP4 consists of 183 amino acids (after final cleavage of 18 amino acids at the N-terminus) in a single polypeptide chain [7,8]. The structure was investigated by X-ray crystallography and revealed the following characteristics: an N-terminal coil, a C-terminal α-helix and subsequent coil region, and a β-barrel core [9]. Within that characteristic β-barrel core, RBP4 can specifically bind one molecule of retinol to transport it through the bloodstream. Due to the ability to bind the lipophilic molecule retinol, RBP4 belongs to the protein family of lipocalins [10,11]. With the specific retinol transport function, RBP4 is a crucial regulator of retinoid homeostasis.

The major part of retinoids (~80%) is stored in the liver, whereas other organs usually contain some, but overall much lower amounts when compared to the liver [12]. Consistent with this observation is also the RBP4 gene expression profile pattern: its transcript is found in many organs and tissues, such as liver, white and brown adipose tissues, kidney, lung, retinal pigment epithelium (RPE), testes, brain, and choroid plexus, of which the expression in liver is by far the highest [12,13,14,15,16,17,18,19,20].

Genomic elements in the 5’ flanking region of the human *RBP4* gene that are accountable for the high hepatic transcription have been identified, although the exact characteristics of these regulators remain to be clarified [13,21,22]. The transcriptional regulation was shown to be mediated by a multiprotein complex, comprising high mobility group A1, protein-associated splicing factor, and steroidogenic factor 1, among others, and has been additionally described as inducible by cyclic AMP stimulation [23,24]. Notably, cyclic AMP had been identified earlier as an inductor of *Rbp4* mRNA expression in murine hepatocytes [25]. Another study investigated the effects of glucagon and fasting of mice and found both of them to induce hepatic *Rbp4* mRNA expression [23,24]. The induction of *Rbp4* mRNA expression upon fasting was also detectable in mice lacking one of the hepatic fasting master-regulators, peroxisome proliferator-activated receptor α (PPARα) [26], indicating a PPARα-independent mechanism [27].

Changes in the dietary supply of vitamin A—in form of sufficiency, depletion, and repletion—were shown not to alter mRNA expression or protein translation of RBP4 [28]. Notably, the vitamin A derivatives all-*trans* retinoic acid (atRA) and 9-*cis* RA were found to be potent inducers of *Rbp4* gene expression in vitro (Hepa 1–6 cells) and in vivo (murine liver) [29]. Another study investigated the effects of atRA treatment, which lowered mRNA expression of *Rbp4* in adipose tissue but not in the liver, while repressing hepatic RBP4 protein and inducing serum RBP4 levels [30].

Furthermore, the translation of *Rbp4* mRNA was increased upon acute re-feeding [31]. The same study found that this was dependent on ‘mechanistic target of rapamycin in complex 1’ (mTORC1) activation. Accordingly, using an mTORC1 inhibitor suppressed the regulation by re-feeding and inhibited *Rbp4* translation [31]. Other translational regulators of *Rbp4* have not been identified so far.

Human RBP4 was found to be shortened by one or both leucine amino acid residues at the C-terminus in patients suffering from chronic renal failure [32]. In this study, it was hypothesized that the truncated RBP4 protein is generated within the hepatocyte, released into the bloodstream, and then, due to the malfunctions of the kidney, cannot be filtrated [32]. This is in line with the finding that truncated RBP4 proteins are detected in a number of diseases of the kidney but not of the liver [33]. The functional role of truncated RBP4 is thus far unknown. 

## 2. RBP4 Secretion

### 2.1. Source of Circulating RBP4

RPB4 in humans circulates at 2–3 µM, while in the circulation of mice, it amounts to ~1 µM [34]. Most of this represents retinol-loaded holo-RBP4, in line with the notion that RBP4 and retinol concentrations in serum match well [34]. Although dietary vitamin A intake may vary, RBP4 concentrations, and with that retinol concentrations, are usually maintained within this narrow range under physiological conditions. The aforementioned large number of tissues that express *Rbp4* raises the question which of these organs contribute, and to what extent, to circulating RBP4 concentrations and whether a tissue-specific origin could render RBP4 exhibiting distinct properties. Strikingly, it was found that hepatocyte-specific deletion of RBP4 in mice resulted in undetectable RBP4 levels in serum, suggesting that, at least in mice, hepatocytes are the sole origin for circulating RBP4 [35]. This also argues against the hypothesis that elevated RBP4 in the circulation of obese/insulin resistant subjects [36] is due to increased RBP4 secretion by adipocytes [37,38]. Liver-specific RBP4 overexpression readily translated into elevated RBP4 levels in the circulation of mice [39,40], further supporting the pivotal role of the liver in determining blood RBP4 levels. Moreover, patients with certain hepatic diseases such as liver cirrhosis, interfering with the hepatic biosynthetic capacity, were found to have reduced circulating RBP4 levels [41]. In fact, RBP4 behaves as a negative acute phase reactant and is reduced in critically ill patients with or without sepsis [42,43]. Another study investigated the effects of elevated adipocyte-specific expression of RBP4 in a transgenic mouse model [44]. Consistently, although RBP4 expression in adipocytes was increased, this did not translate into elevated serum RBP4 concentrations of standard chow-fed mice. Interestingly, adipocytes in vitro, as well as fat pad explants, were shown to secrete measurable amounts of RBP4 into the culture media [18,35,45]. Thus, although extrahepatic tissues may contribute to circulating RBP4 levels, evident studies are sparse. However, support for this is provided by studies in which RBP4 was overexpressed specifically in muscle cells and where the loss of circulating RBP4 due to global RBP4 knockout could be reversed [46,47]. This indicates that other tissues, here exemplified by the muscle, can contribute to circulating RBP4 concentrations under specific circumstances—at least by using robust transgenic overexpression. Taken together, circulating RBP4 seems to derive exclusively from the liver in a healthy organism. Nevertheless, it remains to be investigated whether other organs may contribute to circulating RBP4 under pathological states. 

RBP4 is circulating not only within the bloodstream, but also in the cerebrospinal fluid [14,15]. Here, the choroid plexus is thought to be the site of origin [48].

### 2.2. Hepatic Retinoid Storage and Mobilization 

All vitamin A is derived from nutritional uptake [1]. The ingested lipids and retinyl esters are packed into chylomicrons within the intestine [49,50]. These chylomicrons reach the organs via the lymphatic system. Within the capillaries, chylomicrons release parts of their stored lipids and retinoids via the action of lipoprotein lipase (LPL) that induces the hydrolysis of esters such as retinyl esters [51]. The significant role of this lipase becomes intelligible when RBP4 is absent (RBP4 knockout mice), and LPL activity is the major source for the maintenance of retinoid homeostasis through depletion of chylomicron and other lipoproteins from incorporated retinyl esters [52]. Thereafter, the chylomicron remnants reach the liver where the majority of the organism’s retinoids are stored in form of retinyl esters in hepatic stellate cells [53]. Chylomicrons are unpacked within hepatocytes and release its content. To this date it is not completely understood how retinol is transported from hepatocytes to hepatic stellate cells that store retinyl esters together with other lipophilic molecules within their characteristic lipid droplets [54]. It was shown using a RBP4-deficient mouse model, that the storage of retinyl esters in liver was unaltered, pin-pointing that the transport between both hepatic cell types does not depend on RBP4’s action [55]. Instead, cellular retinol binding protein 1 (CRBP1, or also RBP1 as the *Rbp1* gene product) was shown to be required for adequate retinyl ester accumulation in stellate cells [56].

For storage, retinol is transacylated by lecithin retinol acyltransferase (LRAT) to form retinyl esters, with retinyl palmitate as the predominant product [57,58,59,60]. Retinol can be released from stored retinyl esters by hydrolysis through retinyl ester hydrolases (REH), marking the first step in the mobilization of retinol from the liver [61,62]. Pivotal for hepatic retinol mobilization is RBP4 as its deletion results in highly increased retinoid content in the liver and, correspondingly, in a drastic reduction of serum retinol levels to about a tenth [34,55]. This dependency also became apparent in a study that utilized acute hepatic RBP4 overexpression to induce retinol mobilization from the liver, which led not only to increased RBP4 and retinol levels in serum, but also decreased hepatic retinyl ester stores [40]. These findings are in accordance with observations of another study in which the secretion of RBP4 was found to be tightly regulated by the presence of retinol, concluding that RBP4 is only secreted from hepatocytes when bound to its ligand [63]. In vitro studies showed that the absence of retinol in primary hepatocytes prevented, whereas a subsequent replenishment increased, RBP4 secretion [64]. Hepatic RBP4 secretion in vivo was diminished through the dietary depletion of retinoids. Concomitantly, in the same study, the abundance of RBP4 protein in liver was increased, indicative of impaired mobilization and consequently RBP4 accumulation in liver [65].

**Figure 1:**
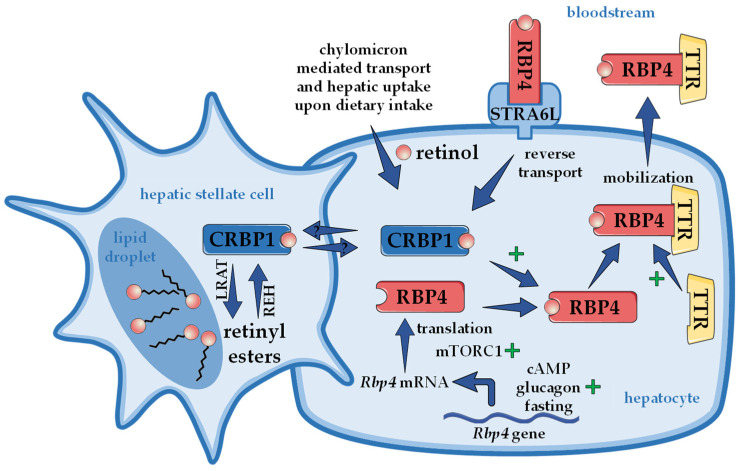
**Hepatic retinoid storage and mobilization.** Upon dietary ingestion, chylomicron-incorporated retinyl esters enter the liver as retinol and are stored as retinyl esters in specialized hepatic stellate cells. Retinyl esters are thereby formed by lecithin retinol acyltransferase (LRAT). How retinol is transferred between hepatocyte and hepatic stellate cells is largely unknown but likely involves cellular retinol binding protein 1 (CRBP1). In order to mobilize retinol, retinyl esters are hydrolyzed by retinyl ester hydrolases (REH). Within the hepatocyte, retinol binds to retinol binding protein 4 (RBP4), which forms a complex with transthyretin (TTR) and is then secreted into the bloodstream. The presence of retinol as well as TTR enhances secretion of the complex. The specific RBP4 receptor in the liver ‘stimulated by retinoic acid 6-like’ (STRA6L, also named RBPR2) is thought to mediate reverse transport of retinol, allowing a futile cycle of retinol between the circulation and liver. *Rbp4* mRNA levels are upregulated in the presence of cyclic AMP (cAMP), glucagon, and during fasting, while the translation is enhanced through ‘mechanistic target of rapamycin in complex 1’ (mTORC1). At least in mice, circulating RBP4 derives exclusively from liver, rendering RBP4 a hepatokine.

When RBP4 was first discovered, it was already realized that circulating RBP4 is bound to another protein that was named prealbumin, later transthyretin (TTR), with a molecular weight of 55 kDa, when in its more stable tetrameric form [6,66,67]. Retinol-loaded RBP4 binds to TTR tetramers within the endoplasmic reticulum of hepatocytes, before the complex is secreted into the bloodstream [68]. TTR functions within the retinoid homeostasis by protecting the circulating complex—that is, through the TTR bond now characterized by a higher molecular weight—from catabolism and renal filtration in order to ensure the delivery of retinol to extrahepatic tissues [69]. Interestingly, TTR also serves as a transporter for thyroid hormones [70]. Mice lacking TTR show an accumulation of RBP4 in the liver, indicating that TTR enhances the secretion of the RBP4/retinol complex [71]. The same phenomenon was found during an acute and liver-specific TTR depletion [39]. Interestingly, although RBP4 secretion appears to depend on TTR, a knockout of TTR does not completely inhibit RBP4 secretion, as it was still detectable in the serum of TTR knockout mice. RBP4 secretion was also observed in TTR-deficient primary hepatocytes [69]. Consistently, TTR knockout mice appear not to lack retinoids in relevant organs, such as the liver and eye [71]. This supports the notion that TTR’s function in this complex is limited to stabilizing the RBP4/retinol complex, without having major implications for the systemic retinoid homeostasis. Furthermore, combined with the fact that the absence of functional RBP4 (knockout mice) leads to reduced but detectable serum retinol levels, this suggests that other alternative transport pathways have evolved to ensure retinoid homeostasis. Alternative retinol transport by albumin has been suggested [40], but retinyl esters were also shown to be carried within lipoproteins, such as liver-derived very low-density lipoprotein and low-density lipoprotein [72]. Another possible pathway of retinol delivery, when RBP4’s function is compromised, is the afore-described delivery of retinoids via chylomicrons [55,73]. In any case, the importance of vitamin A and its physiological functions become very evident by these studies, providing insights in the tightly regulated retinol transport by RBP4 and that situations where RBP4 cannot fulfill its function trigger compensatory delivery pathways. An overview of hepatic retinol mobilization orchestrated by RBP4 is presented in Figure 1.

## 3. RBP4 Receptors 

**Figure 2:**
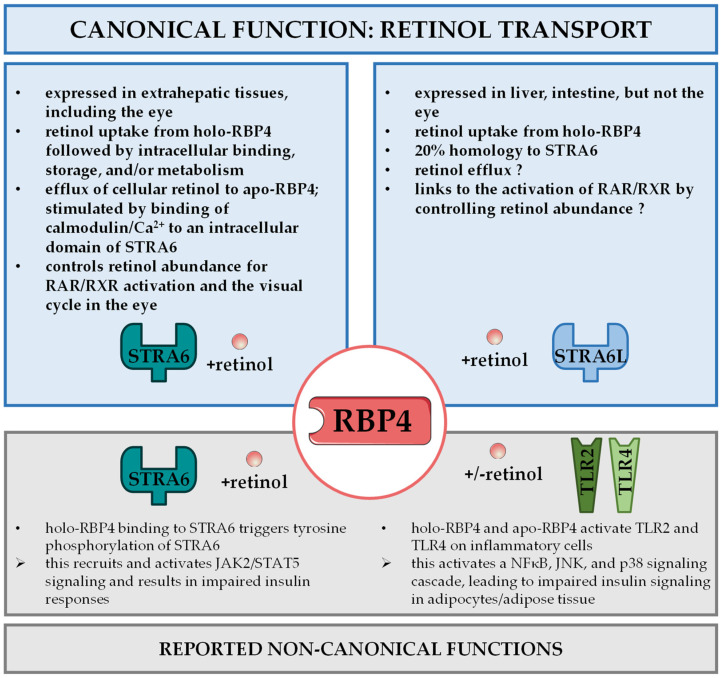
**RBP4 receptor interactions.** Distinct receptor proteins mediate canonical and non-canonical functions of RBP4. ‘Stimulated by retinoic acid 6’ (STRA6) in extrahepatic tissues and ‘stimulated by retinoic acid 6 like’ (STRA6L, also known as RBPR2) in liver and intestine are involved in retinol uptake and its coupling to RAR/RXR signaling in target cells and the visual cycle in the eye, referred to as canonical functions of RBP4 (top panel). STRA6 was also reported to activate janus kinase 2 (JAK2)/signal transducer and activator of transcription 5 (STAT5) signaling to impair insulin signaling. RBP4 recognition by Toll-like receptors 2 and 4 (TLR2/TLR4) was shown to induce a signaling cascade involving nuclear factor κB (NFκB), c-jun N-terminal kinases (JNK), and p38 that impedes on insulin sensitivity. Both mechanisms are designated as non-canonical functions of retinol (bottom panel).

Once secreted into the bloodstream, the complex comprising retinol, RBP4, and TTR is distributed to extrahepatic tissues. On the surface of target cells ‘stimulated by retinoic acid 6’ (STRA6) has been identified as RBP4’s main receptor to induce the uptake of retinol [74], as shown in Figure 2. Notably, STRA6 was known more than a decade ago as a likely membrane protein that was inducible by atRA even before the discovery that RBP4 can actually bind to it and promote the transport of retinol across the cell membrane [74,75]. In accordance with this function, STRA6 is most highly expressed in extrahepatic tissues that require substantial retinol uptake for their intrinsic function. Among these cell types and organs are RPE, female reproductive organs, testis, brain, and kidney [20,76]. Due to the significant role of retinoids in embryonic development, the expression of STRA6 is also high at certain stages of embryogenesis [75].

STRA6 displays a structural feature that allows the β-barrel core of RBP4 to access a specific loop in the receptor to facilitate retinol release [77]. A crystallographic study of zebrafish STRA6 gave new insights when one intramembrane and nine transmembrane helices were identified, as well as their complex dimeric assembly that gives access to a lipophilic cleft, which reaches within the membrane and plays a crucial part in transporting retinol across the lipid double layer [78]. Due to the 3D structure, it is thought that retinol-containing holo-RBP4 has to be isolated from TTR to provide the needed space for interaction with STRA6, and, moreover, that free retinol within the membrane can inhibit STRA6’s function [79]. After extracellular binding of RBP4 to STRA6, the receptor mediates retinol transport through the membrane and intracellular binding to cellular retinol binding protein 1 (CRBP1). Subsequent esterification of retinol and storage of retinyl esters, which frees CRBP1 to potentially accept another molecule of retinol, is induced by the activity of LRAT [79,80]. Interestingly, STRA6 is not only the key mediator of retinol uptake but was also shown to mediate retinol export to load retinol-free apo-RBP4 in cell culture experiments. This mechanism could protect cells from excessive retinoid activity [80], further supported by the inducibility of STRA6 expression by atRA [75], which likely forms a negative feedback mechanism.

Interestingly, STRA6 can bind calmodulin, which has been shown to promote, in association with Ca^2+^, extracellular binding of apo-RBP4 and retinol export in vitro [81]. The deletion of STRA6 in mice showed a phenotype where, although viable, the visual capability and the ocular morphology were impaired due to decreased retinoid contents in the RPE and retina [48,82]. Thus, STRA6 is a crucial cell surface receptor that regulates retinoid homeostasis by induction of retinol uptake. With the ability to transport retinol bi-directionally in vitro and to promote the coupling to specific carrier proteins, the potential to balance the physiologically tightly regulated retinoid homeostasis in extrahepatic tissues through STRA6 is of enormous interest [83]. The physiologic relevance of STRA6-mediated retinol efflux, although observed in vitro [40,79,80,81,83], is unclear.

To some surprise, the phenotypes of murine STRA6 loss-of-function models showed less severe phenotypes than what has been observed in humans with STRA6 mutations. These mutations are associated with multiple pathologies and referred to as the Matthew–Wood syndrome. Among the detected abnormalities of this syndrome are anophthalmia, congenital heart defects, diaphragmatic hernia, alveolar capillary dysplasia, lung hypoplasia, and intellectual disability [84,85,86].

In the liver, STRA6 is barely expressed [76]. Instead, ‘stimulated by retinoic acid 6-like’ (STRA6L), also called RBP4 receptor 2 (RBPR2), shows robust expression in the liver and some other organs such as the small intestine and colon [87]. STRA6 and STRA6L show a structural homology of about 20% [87]. In addition to structure, functions also appear to differ between both receptors. STRA6L has been shown to promote uptake of retinol, but while STRA6 can induce retinol export in vitro, this has yet to be shown for STRA6L [87]. STRA6L’s function can be inhibited by retinol and atRA, and its expression is not induced, rather downregulated by atRA [87]. In addition, STRA6L was shown to be required for normal development and functionality of the zebrafish eye [88,89,90]. Strikingly, STRA6L is not expressed in the zebrafish’s eye itself, indicating that STRA6L functions in this context through alterations in systemic retinoid homeostasis [89]. In summary, very little is known about STRA6L and its implications in retinoid homeostasis to date. Canonical functions of RBP4 via these two receptors are summarized in Figure 2, and both receptors will be of great interest to complete our understanding of the multifaceted functions of RBP4. In addition to receptor-mediated uptake of retinol from RBP4, receptor-independent diffusions of retinol through the cell membrane may also occur, especially upon RBP4 receptor dysfunction [91]. 

## 4. Renal Filtration and Recycling of RBP4

After delivering retinol to target cells, RBP4 remains in the bloodstream without being bound to either retinol or TTR. Having lost these partners, RBP4 is a target of renal filtration. Nevertheless, within the proximal renal tubule, the megalin-cubilin receptor complex initiates almost complete reabsorption of RBP4 and therefore its recycling, as concluded from a kidney-specific megalin knockout mouse model [92,93]. This highly effective recycling process renders increased RBP4 levels in urine as a powerful marker to diagnose proximal tubule malfunctions [94]. The aforementioned kidney-specific megalin knockout model draws new attention to the rather unexplored role of the kidney in vitamin A metabolism. In this study, not only the loss of RBP4 via the urine but, rather unexpectedly, also the loss of retinol were observed alongside with a robust reduction of the retinoid content in liver [93]. These observations raise the questions of how and to what extent recycled RBP4 is loaded with retinol in renal tubules and whether it forms a complex with TTR before re-entering the circulation. 

## 5. RBP4: A Carrier for Non-Retinoid Ligands?

Ongoing research on RBP4 proposes that this protein can transport other molecules aside from retinol. In crystallographic studies, expression-host-derived oleic and linoleic acid were found in RBP4’s binding pocket [95]. Both RBP4 crystal structures with the protein data bank IDs 2WQ9 [95] and 2WR6 [96] suggest that RBP4 may also be capable of transporting certain fatty acids. Accordingly, another study found fatty acids within the crystal structure of human retinol-free RBP4 isolated from serum, urine, and amniotic fluid [97]. Several fatty acids showed the capability of ^3^H-retinol replacement in the binding pocket of human RBP4, providing another hint to the fatty acid binding and possible transporting function of RBP4 [98].

## 6. RBP4 in Health and Disease

The following chapter describes the most important findings of RBP4’s contributions to health and disease, and Table 1 summarizes these findings regarding the involved organ systems and physiological processes.

### 6.1. RBP4 and Vision

Some of the most prominent consequences of vitamin A deficiency are in regard to vision: night blindness and full blindness [117], as well as impaired vision in newborns when vitamin A intake was not sufficient during pregnancy [118] have been described. Therefore, it is not surprising that a dysfunctional retinol transport by RBP4 also has consequences on the eye and its functionality. It was found that especially the eye cannot efficiently take up retinol in the absence of RBP4 [119], suggesting that adequate levels of 11-*cis* retinaldehyde in the eye as the light-sensitive chromophore of the rhodopsin complex [120] require RBP4-mediated retinol uptake.

Interestingly, RBP4 deletion in mice on a mixed genetic 129xC57BL/6J background and fed a normal chow diet induced visual defects (retina dysfunction and impaired visual acuity) only in the first four to five months after birth [55]. However, when fed a vitamin A-deficient diet, these mice were not able to balance the malfunctions after a few months and showed a further deterioration of their ocular function [55]. The adaptation to RBP4 deletion was reported to be slow in comparison to those of other organs since the eye cannot efficiently change retinoid supply to alternative pathways [119]. Rescue studies in RBP4 knockout mice, by either muscle-specific expression of RBP4 [46] or by inserting a human RBP4 open reading frame in the mouse *Rbp4* locus [111], were not only able to replenish serum RBP4 and serum retinol levels but also reversed the visual insufficiencies. Another study with C57BL/6 mice with a global RBP4 knockout and vitamin A-sufficient chow observed even more severe abnormalities: the ocular physiology was impaired in many of its structures [110]. Moreover, defects were reported to not have reversed or improved by 40 weeks of age [110]. These studies underline the importance of a functional vitamin A homeostasis especially for vision and its dependence on RBP4. Of note, a muscle-specific overexpression of RBP4 was also reported to cause progressive retinal degradation, of which inflammatory processes have been suggested to play a causative role, rather than alterations in retinoids homeostasis [106,121].

Mice lacking the RBP4 cell surface receptor STRA6 displayed an ocular phenotype, including changed morphology and anatomy of the eye, but lacked major anomalies in other organ systems [48,82,91,122,123]. Thus, at least in murine models, most organs except the eye appear to be less dependent on RBP4-mediated retinol delivery, in particular when sufficient nutritional uptake of retinoids is warranted.

Most of the identified mutations of *RBP4* in humans are linked to impaired vision. The compound heterozygous mutations p.I59N and p.G93D (I41N and G74D in the cleaved RBP4 protein) were found to cause night blindness and modest retinal dystrophy without effects on growth [112,115]. The observed absence of RBP4 and the strong reduction in circulating retinol is thought to be due to an increased instability of the RBP4/retinol complex [124]. Autosomal dominant congenital eye malformations that include microphthalmia, anophthalmia, and coloboma disease were found to be caused through heterozygous p.A73T and p.A75T mutations [113]. In these mutations, the maternal penetrance was higher than the paternal penetrance. The study revealed that these mutations in *RBP4* led to a much less effective binding of its substrate retinol while the affinity of the mutated RBP4 to STRA6 was highly increased. This in turn resulted in a disturbance of vitamin A metabolism, as its receptor preferably bound to the mutated and therefore empty carrier and thus did not allow for retinol uptake [113]. Although very rare, a bi-allelic c.248 + 1G > A mutation that led to extremely low RBP4 serum levels caused retinal dystrophy and ocular coloboma [114]. Another pathology was associated with the homozygous splice site variant c.11 + 1G > A of *RBP4* [104]. Here, aside from the diminished RBP4 levels in serum, retinal dystrophies (progressive and severe autosomal recessive retinitis pigmentosa) and developmental abnormalities were identified. The homozygous c.67 C > T *RBP4* mutation produces a truncated RBP4 protein due to the introduction of an early stop codon. The encoded protein is predicted to be non-functional and the mutation is linked to the development of retinitis pigmentosa [116]. 

### 6.2. RBP4 and Embryonic Development

Vitamin A is highly relevant for embryonic development, which is also reflected in the finding that expression of the RBP4 receptor STRA6 is strongly induced during certain stages of embryogenesis [20,76]. Until now, no human *RBP4* null mutations have been identified [125]. This may point towards the incapability of the human embryos to survive without functional RBP4 expression. In global RBP4 knockout mice, where RBP4 remains undetectable and retinol levels were reduced to about 12%, their offspring were born viable [55] and with only temporary, minor phenotypes regarding heart development [103]. However, a dietary vitamin A deficiency together with a loss of RBP4 during pregnancy had major consequences for the embryo: reduced size, malformations in the midfacial region, and exencephaly were observed [73]. These phenotypes were also found within the severe fetal vitamin A-deficiency syndrome and mouse models of retinoic acid receptor (RAR) [126] and retinoid X receptor (RXR) [127,128,129] deficiencies [130], the primary targets of atRA and derivatives that mediate most of the known effects of vitamin A.

Overall, the sensitivity of rodents and humans to alterations in retinoid homeostasis and vitamin A availability may differ substantially, as humans are much more susceptible to retinoid toxicity because of uncontrolled retinoid diffusion [131], which may account for developmental effects of certain human RBP4 mutations described in Section 6.1.

### 6.3. RBP4 and Insulin Sensitivity

It was shown more than 20 years ago that type 2 diabetic patients exhibited increased levels of RBP4 in their blood [132,133]. In 2005, an important study sparked new interest in the relationship between RBP4 and insulin resistance, the pathophysiological driver to type 2 diabetes. It was shown that *Rbp4* expression is upregulated in adipose tissue of obese and diabetic mice and humans, while the insulin-sensitizing thiazolidinediones lowered elevated RBP4 serum levels [36]. Importantly, overexpression of RBP4 in mice or its administration elicited insulin resistance and diminished glucose tolerance, whereas RBP4 knockout mice showed improved insulin sensitivity [36]. A hypothesis emerged that adipose tissue is the source of elevated RBP4 in serum, which would render RBP4 an adipokine, a signal/protein secreted from adipocytes with systemic effects. This notion was supported by tight correlations between serum RBP4 levels, adipose tissue *RBP4* mRNA expression, visceral fat mass, and insulin resistance in humans [134]. Another study positively correlated serum RBP4 levels with insulin resistance and additionally showed that improving insulin sensitivity through exercise lowers RBP4 levels [135]. However, by now and at least in mice, it is generally accepted that circulating RBP4 is primarily derived from liver and not adipose tissue and as delineated in Section 2.1 [35]. Thus, RBP4 is unlikely to be an adipokine, at least not when compared with specific characteristics of prototypical adipokines such as adiponectin and leptin [37]. Regarding genetic studies in humans, several single-nucleotide polymorphisms of *RBP4* were reported to be associated with metabolic diseases and related risk factors, including the risk of hypertriglyceridemia in Chinese Hans [136], increased serum high density lipoprotein levels [137], risk of gestational diabetes [138], higher body mass index [139], elevated insulin resistance [140], and type 2 diabetes mellitus [141,142,143].

So far, a variety of different mechanisms for how elevated RBP4 in the circulation may interfere with insulin sensitivity have been reported [144]. The initial study showed that RBP4 administration induced expression of phosphoenolpyruvate carboxykinase in the liver, the rate-limiting enzyme of gluconeogenesis and thus contributor to hyperglycemia, and impaired insulin signaling in muscle [36]. The same authors later provided evidence that also inflammatory processes in adipose tissue were involved. They showed that RBP4 affects macrophages, dendritic cells, and CD4^+^ T-cells in adipose tissue, thereby promoting an inflammatory response, impairing insulin signaling and eventually leading to insulin resistance [107,108,145,146]. They presented a mechanism that is independent of not only retinol, but also of the aforementioned RBP4 receptor STRA6 [146]. Instead, Toll-like receptors 2 and 4 (TLR2/4) were activated and induced the secretion of inflammatory cytokines such as interleukin 1 β or tumor necrosis factor α (TNFα) by a signaling cascade that utilizes nuclear factor κB, c-Jun N-terminal kinases and p38 [108,145], summarized in in Figure 2.

Another group suggested an alternative mechanism linking elevated RBP4 to insulin resistance, which was dependent on STRA6 and retinol [147], as depicted in Figure 2. In their model, the RBP4/retinol complex binds to STRA6 and thereby induces phosphorylation of STRA6 close to the C-terminus. This causes Janus kinase 2 (JAK2) activation, which in turn activates signal transducer and activator of transcription 5 (STAT5). Eventually, this upregulates the expression of genes, such as suppressor of cytokine signaling 3, that are known to possess insulin inhibitory effects. The dependence on retinol became even more obvious when it was shown that the transfer from retinol across the membrane to an intercellular acceptor protein is required for a JAK2/STAT5 activation [148], while TTR was shown to be disruptive for retinol transport and an activation of these signaling steps involving STRA6 [149]. Consistently, injecting holo-RBP4 in mice that lacked STRA6 did not activate the JAK2/STAT5 signaling cascade or impair insulin sensitivity [91]. 

A very recent study provided evidence that the growth hormone receptor promoted RBP4 expression and secretion via a phosphorylation of STAT5 in liver [150]. Moreover, they found that STAT5 increased TTR expression via hypoxia-inducible factor-1 α, thereby delaying renal clearance and contributing to elevated RBP4 in the circulation and triggering insulin resistance [150]. How these findings relate to the proposed holo-RBP4/JAK2/STAT5 activation pathway is currently unknown.

RBP4 was also shown to induce β-cell dysfunction [109]. The authors of the study showed that pancreatic *Stra6* expression is especially high in β-cells. It was found that holo-RBP4 inhibits insulin synthesis via a STRA6/JAK2/STAT1/insulin gene enhancer protein-1 cascade as the underlying cause for the age-dependent decrease in glucose-stimulated insulin secretion of C57BL/6J mice with CAG promoter-driven, transgenic overexpression of human RBP4 [109].

Despite the fact that elevated levels of circulating RBP4 in insulin-resistant and type 2 diabetic subjects was reproducibly found in most studies, whether or not RBP4 is indeed causative and the exact mechanisms are still heavily debated in the field [144]. Some discrepancies involve the lack of elevated *Rbp4* expression in the adipose tissue of obese patients reported by some studies [151] and the dependence of serum RBP4 concentrations on renal filtration, which is known to decline with type 2 diabetes and may actually be the underlying reason for elevated RBP4 levels in these subjects [152,153]. Other studies did not observe preserved insulin sensitivity and glucose tolerance in RBP4-deficient mice when challenged by a high-fat diet [105]. Moreover, neither acute nor long-term liver-specific overexpression of murine RBP4 showed impaired insulin sensitivity and glucose tolerance, although circulating RBP4 levels were increased to the same extent as observed in insulin-resistant states [39,40], suggesting that at least liver-secreted RBP4 is not necessarily inducing insulin resistance. More research is needed to fully understand RBP4’s involvement in metabolic control and to dissect the underlying mechanisms. 

### 6.4. RBP4 and Adipose Tissue Lipolysis

Adipose tissue shows substantial expression of *Rbp4* [18], the function of which, if secreted without reaching the circulation, remains enigmatic. An elegant study investigated mice with adipocyte-specific overexpression of human RBP4 to study its role in this tissue [44]. As described in Section 2.1, adipocyte-restricted overexpression did not translate directly into elevated serum levels. This argues for local effects of ectopic RBP4, working either intracellularly or within the adipose tissue compartment. Interestingly, transgenic mice were less tolerant to glucose, exhibited increased non-esterified fatty acid concentrations in serum and elevated hepatic triglyceride levels, which led to the development of hepatic steatosis [44]. The authors showed that increased lipolysis in adipose tissue contributed to the influx of lipids into the liver, thereby triggering steatosis. If this was mediated by the induction of lipolysis through RBP4 itself or indirectly, via the induction of inflammatory pathways that involve TNFα and other cytokines [44], is currently speculative. Somewhat consistent is the observation that global RBP4 knockout mice showed reduced levels of circulating non-esterified fatty acids, supporting a potential connection between RBP4 and lipolysis [36]. In vitro studies with human adipocytes made observations in favor of a causal link, since RBP4 promoted fatty acid release [154]. Moreover, it was shown that co-culture with macrophages promoted this effect by the secretion of pro-inflammatory cytokines that have been shown to influence lipolysis through a modulation of the insulin-signaling pathway [154]. The here described findings may also directly relate to the notion that RBP4 can bind fatty acids and due to the fact that fatty acid-binding proteins or other fatty acid acceptors such as albumin readily activate fatty acid release [155] via, for instance, mass action.

### 6.5. RBP4 in Cardiovascular and Renal Diseases

RBP4 levels in blood are associated with cardiovascular diseases such as hypertension [156,157,158,159]. Functionally, a RBP4 loss-of-function mouse model showed lower blood pressure, enhanced (ex vivo determined) carbachol-induced vasodilatation of carotid arteries, some protection from angiotensin 2-induced hypertension, and reduced cardiac hypertrophy [100]. Mice with transgenic overexpression of RBP4 showed the opposite effects: blood pressure was increased, while ex vivo vasodilatation was reduced. This study’s results indicate that RBP4 is an active contributor to blood pressure regulation [100]. Similar to diabetic nephropathy, hypertension-induced kidney dysfunction may also be responsible for elevated serum levels in cardiovascular patients [160].

Coronary artery disease (CAD) was also found to be associated with increased RBP4 levels [161,162,163,164,165]. Consistently, human *RBP4* single-nucleotide polymorphisms were linked with cardiovascular risk factors [166]. It was shown that RBP4 induces inflammatory processes in endothelial cells [167] and promotes hyperinsulinism-induced vascular smooth muscle cell proliferation and migration [168]. Nonetheless, other studies could not conclude a positive correlation between RBP4 and ischemic cardiac events [169,170]. A recent meta-analysis that included about 7000 participants found no correlation between CAD and RBP4 levels, whereas patients suffering from complications such as hyperinsulinemia and subclinical hypothyroidism were shown to be more likely to exhibit higher RBP4 concentrations in their circulation [171]. In mice, RBP4 was shown to promote cardiac injury after myocardial infarction via inducing cardiomyocyte pyroptosis through an interaction with NLR family pyrin domain containing-3 [101].

Not unexpectedly, chronic kidney disease (CKD) can also increase RBP4 in the circulation [172]. Experimentally, a 5/6 nephrectomy in mice increased concentrations of retinol and RBP4 in blood, which promoted the expression of G protein-coupled receptor-68 in circulating monocytes [173]. Cardiac infiltration of these monocytes under CKD conditions may exacerbate inflammation and fibrosis of the heart.

### 6.6. RBP4 and Non-Shivering Thermogenesis during Cold Adaptation

Murine and human circulating RBP4 levels increase upon exposure to cold [102], suggesting that increased retinol delivery to peripheral tissues may be involved in the adaptive response that increases non-shivering thermogenesis also in white adipose tissue, referred to as adipose tissue browning. Indeed, the authors of that particular study showed that cold exposure led to increased expression of *Rbp4* in mouse liver but not white or brown adipose tissue and elevated retinol concentrations in blood, indicative of increased mobilization of hepatic retinoid stores. Moreover, cold-exposed mice that lacked RBP4 failed to induce the thermogenic gene expression profile in their inguinal adipose tissue and failed to maintain their body temperature [102]. This was associated with decreased activation of hormone-sensitive lipase, a known contributor of fatty acid release to fuel and activate of uncoupling protein-1 during browning. Interestingly, cold exposure-induced adaptions in brown adipose tissue did not show any dependence on RBP4. Mechanistically, the authors provided evidence that retinol delivery could enhance the oxidative capacity of adipocytes, perhaps via increased expression of thermogenic genes [102]. Whether the aforementioned direct effects of RBP4 on lipolysis may be involved is currently unknown. It is also worth noting that many arctic animals (including the polar bear and seals) store very high amounts of retinoids in the liver [174], which may support a role of hepatic retinol mobilization to promote cold tolerance. Further research is needed to fully understand the relevance of RBP4-mediated retinol transport in the physiological context of cold adaptation.

### 6.7. RBP4 and Neuropathology

To date, only few studies implicate RBP4 in the physiology of the brain and the central nervous system. Although *Rbp4* gene expression was found in many brain regions [12,13,14,15,16,17], its exact local function remains yet to be discovered. Nevertheless, it was found that mice lacking RBP4 behave more anxiously and show reduced locomotor activity. It was hypothesized that loss of functional RBP4 influences the behavior of these mice by promoting anatomical abnormalities, such as neuronal loss and gliosis in the cortex and hippocampus, and reduced proliferating neuroblasts in the subventricular zone [99]. These findings also suggest that other phenotypes of global RBP4 knockout mice could be, at least partially, due to central functions of the protein. Mice lacking TTR showed partially but not completely overlapping behavioral and neuropathological alterations [99].

## 7. RBP4 as Therapeutic Target?

RBP4 coordinates retinol transport and mobilization of hepatic retinoid stores. Although highly homeostatic, certain conditions, delineated in detail in the previous chapters, may indeed benefit from a pharmacological reduction of RBP4 levels in the circulation or diminished retinol delivery that may associate with reduced activation of RAR/RXR-dependent pathways.

Fenretinide, a synthetic derivative of retinoic acid, has antitumoral potency due to its antiproliferative and apoptotic properties in some tumor cells [175]. Fenretinide also interferes with RBP4: it binds RBP4 in the liver and hinders its secretion [176,177]. This inhibitory effect on RBP4 is enhanced by fenretinide being a steric obstacle for TTR binding and thereby promoting renal clearance of RBP4 [176,177,178]. Both mechanisms synergize in lowering the serum levels of RBP4 and thereby the concentration of circulating retinol [179], which, at least in mice, is also dependent on the presence of LRAT [180]. Fenretinide also disturbs the formation of vitamin A from dietary β-carotene by inhibition of β-carotene oxygenase 1 and thereby affects carotenoid partitioning in mice [181]. In addition to being teratogenic, like virtually all retinoid ligands in humans, fenretinide can deteriorate the visual adaption to darkness [182]. 

Fenretinide was also investigated for its metabolic effects in mice. It was observed that mice fed a high-fat diet and treated for six weeks with this retinoic acid derivative normalized their circulating RBP4 levels to those of standard chow diet-fed littermates [36]. Both insulin sensitivity and glucose tolerance were improved by the administration of fenretinide, which is in support of the notion that a RBP4-lowering treatment also exerts metabolically beneficial effects. Furthermore, long-term treatment with fenretinide protected mice from diet-induced obesity, insulin resistance, and hepatic steatosis [183]. Surprisingly, rather similar beneficial effects were observed when treating mice that lacked RBP4, suggesting that fenretinide, at least in part, improves metabolic control independently of lowering circulating RBP4 levels. RBP4-independent effects of fenretinide are likely due to its direct activating effects on RAR/RXR pathways in metabolically active organs, such as adipose tissue, liver, and certain brain regions [184]. 

Fenretinide was also successfully used in human trials for age-related macular degeneration, where its benefits are thought to be mediated by its anti-angiogenic properties [185]. Other degenerative diseases related to the eye might also benefit from fenretinide since it also appears to hinder the accumulation of lipofuscin bisretinoids in the RPE [186].

Another compound to target RBP4 is the non-retinoid ligand A1120. This molecule interrupts the binding of TTR to RBP4 and thereby reduces circulating levels of RBP4 and retinol [105]. The administration of A1120 to high-fat-diet-fed mice led to a normalization of RBP4 levels, but in contrast to fenretinide, did not improve metabolic parameters [105]. These findings are in further support of the notion that RBP4-lowering per se does not necessarily improve insulin and glucose homeostasis in high-fat diet-fed mice. However, its non-retinoid structure, potentially conferring a more applicable safety profile, may allow for new indications including ocular degenerative diseases such as Stargardt macular dystrophy [187,188]. Interestingly, a recent study engineered a RBP4 protein scaffold that interacts with human RBP4 in an A1120-dependent manner [189]. This artificial system could have a wide array of pharmacological implications and was used by the authors to regulate the activity of primary human chimeric antigen receptor T-cells in vitro [189].

The compound BPN-14136 is another a non-retinoid ligand of RBP4 and a potent suppressor of the RBP4/TTR interaction, decreasing RBP4 levels in the circulation of mice [190]. The compound might have a therapeutic potential to treat atrophic age-related macular degeneration and Stargardt disease [190,191]. Moreover, BPN-14136 administration to mice that overexpressed human RBP4 specifically in adipocytes partially prevented diet-induced obesity and hepatic steatosis, most likely via lowering circulating RBP4 levels [98]. Novel non-retinoid structures that bind RBP4 and inhibit the RBP4/TTR interaction, potently lowering RBP4 levels in mice, have been identified and include phenylpyrrolidine derivatives [192].

Indirect strategies to target RBP4 are so-called ‘transthyretin tetramer kinetic stabilizers’. Instead of being ligands for RBP4, these small molecules bind TTR and stabilize its tetrameric assembly [193], thereby also lowering circulating RBP4 levels in mice. As these compounds prevent TTR aggregation in a gel-based assay in vitro, they might reduce the aggregation of unliganded TTR monomers in vivo, known to cause TTR amyloidosis and related comorbidities [193]. Of note, compounds that can bind both RBP4 and TTR bi-specifically have also been identified [194].

Taken together, the number of non-retinoid compounds that lower RBP4 in circulation is growing and may lead the way to treating some of the described pathologies. Since all of these compounds reduce the concentration of RBP4 in blood, they are also referred to as RBP4 antagonists.

## 8. Conclusions

RBP4 is the key transport protein to distribute stored retinoids from the liver to extrahepatic tissues. Significant progress has been made in our understanding of its function by characterizing transgenic mouse models and human mutations. Although alternative delivery systems for retinoids can compensate for dysfunctional RBP4, eye development and vision appear particularly reliant on the supply of retinol via RBP4. Still, many questions remain unanswered about the tissue-specific function of RBP4, alternative non-retinoid ligands, and retinol-independent effects of RBP4 on inflammatory pathways, which are likely to be addressed in future studies. These insights will help shape the therapeutic applicability of RBP4-modifying compounds for some of the described pathologies.

## Figures and Tables

**Table 1:** RBP4 mouse models and human mutations and their phenotypes in different organs systems and processes.

Organ System or Process (in Alphabetical Order)	Mouse Model Phenotypes	Human Mutation Phenotypes
adipose tissue lipolysis	lower circulating levels of non-esterified fatty acids in global RBP4 knockout [36] increased circulating levels of non-esterified fatty acids in adipocyte-specific RBP4 overexpression [44]	
behavior and neurological function	decreased locomotor activity, increased anxiety-like behavior, neuronal loss, gliosis in cortex and hippocampus, and reduction in proliferating neuroblasts in subventricular zone in global RBP4 knockout [99]	
cardiovascular regulation	lower blood pressure, partial protection from angiotensin 2-induced hypertension, and reduced cardiac hypertrophy in global RBP4 knockout [100] higher blood pressure in muscle-specific RBP4 overexpression [100] protection from cardiac remodeling and cardiac dysfunction after acute myocardial infarction by cardiac-specific RBP4 knockdown [101]	
cold tolerance	lower core body temperature, reduced thermogenic activation, and diminished hormone-sensitive lipase activation in subcutaneous white adipose tissue upon cold exposure in global RBP4 knockout [102]	
embryonic development	viable embryos with mild and temporary developmental heart abnormalities in global RBP4 knockout [103] vitamin A deficiency before and during pregnancy leads to severe embryonic malformations (smaller size, undetectable or abnormal midfacial regions and forelimbs, and exencephaly) in global RBP4 knockout [73]	developmental abnormalities in homozygous c.11 + 1G > A mutation [104]
insulin sensitivity and glucose tolerance	increased insulin sensitivity in global RBP4 knockout [36] insulin resistance at 12 weeks of age in muscle-specific overexpression of RBP4 [36] no effect on insulin sensitivity and glucose tolerance (normal chow and high-fat diet) in global RBP4 knockout [105] glucose tolerance not impaired in acute liver-specific RBP4 overexpression [40] no effect of muscle-specific RBP4 overexpression on serum insulin levels and insulin sensitivity [106] improved insulin responses and lower adipose tissue inflammation and CD4^+^ T-cell activation in global RBP4 knockout (on normal chow and high-fat diet; analyzed after feeding low vitamin A diet for 4–5 generations prior to characterization) [107] impaired glucose tolerance and insulin sensitivity and increased adipose tissue inflammation in muscle-specific RBP4 overexpression [107,108] glucose intolerance in adipocyte-specific RBP4 overexpression [44] no alterations in insulin sensitivity or glucose tolerance on control or high-fat/high-sucrose diet in hepatocyte-specific RBP4 knockout [35] insulin response and glucose tolerance not impaired (on normal chow and high-fat diet) in long term liver-specific RBP4 overexpression [39] decreased insulin sensitivity and glucose tolerance through dynamic pancreatic β-cell dysfunction in CAG promoter driven RBP4 transgenic mice [109]	
liver fat	hepatic steatosis and increased uptake of non-esterified fatty acids and elevated gluconeogenic gene expression (when fed high-fat diet) in liver by adipocyte-specific overexpression of human RBP4 [44]	
retinoid homeostasis	circulating retinol levels decrease by ~90% in global RBP4 knockout [55] increased hepatic retinol and retinyl ester content at the age of 5 months in global RBP4 knockout [55] rescue of RBP4 and retinol serum levels when RBP4 was overexpressed in muscle of RBP4-deficient mice [46] increased utilization of lipoprotein-derived retinyl esters in global RBP4 knockout [52] increased serum RBP4 and retinol levels, decreased hepatic retinyl ester levels, and increased RAR activation in the stromal-vascular fraction of epididymal white adipose tissue by acute liver-specific RBP4 overexpression [40] serum retinol levels below detection threshold in global RBP4 knockout [110] increased RBP4 levels in adipose tissue and unaltered circulating RBP4 and retinol levels on normal chow, while increased on high-fat diet in adipocyte-specific RBP4 overexpression [44] serum RBP4 undetectable, circulating retinol levels reduced by more than 93%, and hepatic retinol and retinyl ester content unchanged in hepatocyte-specific RBP4 knockout [35] rescue of plasma RBP4 and retinol levels when human RBP4 open reading frame cloned into mouse *Rbp4* locus of RBP4-deficient mice [111] increased serum RBP4 and retinol levels and unaltered hepatic retinyl ester levels in long-term liver-specific RBP4 overexpression [39]	undetectable serum RBP4 and reduced serum retinol levels in compound heterozygous p.I59N and p.G93D mutation [112] undetectable serum RBP4 levels and reduced serum retinol concentrations in homozygous c.11 + 1G > A mutation [104] poor binding of mutated RBP4 to retinol but higher affinity to STRA6 in heterozygous p.A73T and p.A75T mutation [113] undetectable serum RBP4 levels in bi-allelic c.248 + 1G > A mutation [114]
vision	impaired retinal function and visual acuity after birth which is normalized at the age of 4–5 months when diet is vitamin A sufficient and which cannot be normalized on vitamin A-depleted diet in global RBP4 knockout [55] progressive retinal degeneration in muscle-specific RBP4 overexpression [106] suppression of visual defects when RBP4 was overexpressed in muscle of RBP4-deficient mice [46] severe and persistent visual defects in global RBP4 knockout [110] rescue of retinal function when human RBP4 open reading frame placed into mouse *Rbp4* locus of RBP4-deficient mice [111]	night blindness and modest retinal dystrophy in compound heterozygous p.I59N and p.G93D mutation [112,115] retinal dystrophy in homozygous c.11 + 1G > A mutation [104] autosomal dominant congenital eye malformations (incl. microphthalmia, anophthalmia, and coloboma disease) in heterozygous p.A73T and p.A75T mutation [113] retinal dystrophy and ocular coloboma in bi-allelic c.248 + 1G > A mutation [114] retinitis pigmentosa in homozygous c.67 C > T mutation [116]
